# Simultaneous Presentation of Uveitic Disc Edema and Papilledema in an Adult

**DOI:** 10.1155/2020/8829124

**Published:** 2020-07-08

**Authors:** Chase A. Liaboe, Michael S. Lee, Justin J. Y. Yamanuha

**Affiliations:** University of Minnesota, Department of Ophthalmology and Visual Neurosciences, Phillips Wangensteen Building, 9th Floor, 516 Delaware St SE, Minneapolis, MN 55455, USA

## Abstract

**Purpose:**

To present a case of simultaneous uveitic disc edema and increased intracranial pressure (IICP) in an adult.

**Methods:**

Retrospective case report. *Patients*. A 29-year-old woman affected by bilateral optic disc edema from bilateral posterior uveitis complicated by IICP with papilledema.

**Results:**

Laboratory workup was negative for infectious and systemic inflammatory causes of uveitis. Computed Tomography scan of the chest was negative for Sarcoidosis. Magnetic Resonance Imaging of the brain and orbits revealed a partially empty sella, bilateral posterior globe flattening without optic nerve sheath enhancement, masses, white matter lesions, or meningeal enhancement. Cerebral Magnetic Resonance Venography showed narrowing of the right and left transverse sinuses without thromboses. Prednisone was initiated for the uveitis which improved the vision but caused weight gain. Neurology evaluation with a lumbar puncture in the lateral decubitus position revealed elevated opening pressure and otherwise normal cerebrospinal fluid. Ocular ultrasonography was considered but not available to measure optic nerve sheath diameter. Oral acetazolamide 1000 mg twice daily was started for papilledema as prednisone was tapered. Periocular steroid and intravitreal bevacizumab injections were used for sight threatening cystoid macular edema and choroidal neovascularization, respectively. *Discussion*. While previously described in children, we report the first known case of bilateral uveitic disc edema and papilledema in an adult. This report will discuss recommendations for evaluation of these rarely concurrent conditions and therapy for both uveitic disc edema and papilledema.

## 1. Introduction

Bilateral optic disc edema (ODE) can result from intermediate or posterior uveitis as well as increased intracranial pressure (IICP). Evaluation for the former typically includes optical coherence tomography (OCT), fluorescein and indocyanine green angiography, and laboratory evaluation for infectious and inflammatory causes of uveitis. The workup for possible papilledema includes brain Magnetic Resonance Imaging (MRI) for intracranial masses, hydrocephalus, meningitis, or encephalitis and Cerebral Magnetic Resonance Venography (MRV) for venous sinus thromboses. Ocular ultrasonography by a trained examiner has been shown to safely measure optic nerve sheath diameter [1] and may also be considered in the evaluation for papilledema. A lumbar puncture is also recommended to measure intracranial pressure and analyze the cerebrospinal fluid. A thorough medication history is paramount in these cases as use or tapering of corticosteroids, oral contraceptive pills, tetracycline antibiotics, or Vitamin A retinoid derivatives can cause IICP. Concurrent presentation of bilateral uveitic disc edema and papilledema has been previously reported in children [2, 3]. Treatment of uveitis with systemic corticosteroids in children has also been shown to cause IICP [4], but presentation of bilateral uveitis and papilledema before or after treatment has not been reported in adults.

## 2. Purpose

We present the first reported case of an adult with simultaneous bilateral uveitic disc edema and IICP which worsened with systemic corticosteroids and discuss the need for treatment of both uveitis and papilledema when present concurrently.

## 3. Methods and Patient

A retrospective case report of a single patient is presented herein. The University of Minnesota Institutional Review Board (IRB) exempted review for this deidentified case report.

## 4. Results: Case History

A 29-year-old woman without prior ocular history presented with blurry vision in the left eye (LE). Medical history was significant for viral meningitis (age 11), Hashimoto's thyroiditis, psoriasis, and treated Chlamydia (age 22). Family history was significant for systemic lupus erythematosus in her mother, but there was no family history of uveitis or other eye conditions. There was no recent travel or exposure. Systemic review revealed intermittent headaches, however was negative for other symptoms of systemic disease such as fevers, rashes, mouth sores, genital ulcers, joint pains, or night sweats.

**Figure 1:**
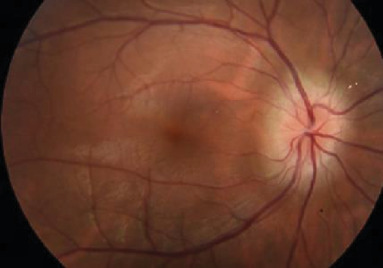
Initial fundus photo of the RE showing optic disc edema.

Initial best corrected visual acuity was 20/20 in the right eye (RE) and 20/500 with pinhole in the left eye (LE). The pupils, intraocular pressure, motility, and alignment were normal. Slit lamp examination showed a quiet anterior segment in the RE and keratic precipitates with 3+ cell and flare in the LE. Dilated funduscopic exam of the RE showed 1+ vitreous cell and optic disc edema (ODE) (Figure 1).

**Figure 2:**
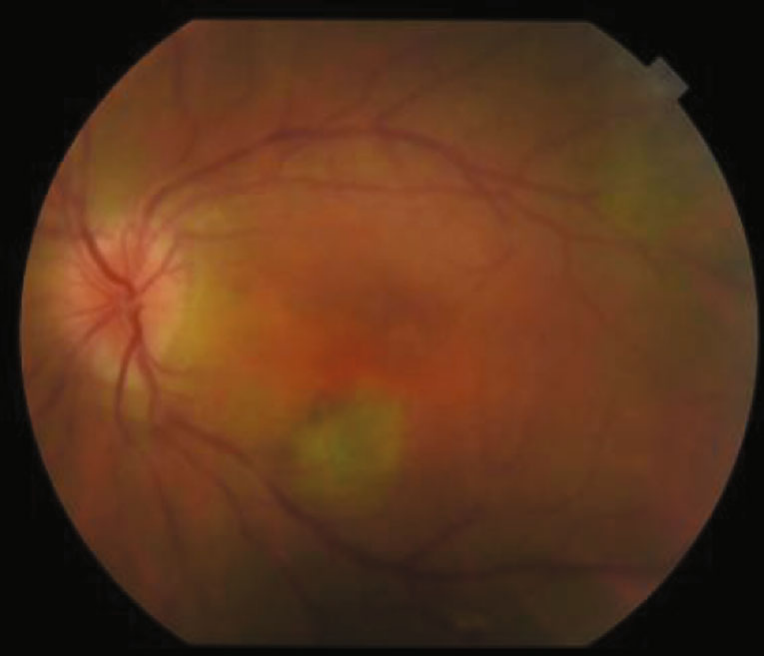
Initial fundus photo of the LE showing disc edema and a gray macular lesion.

Dilated funduscopic exam of the LE showed 3+ vitreous cell, ODE, a gray macular lesion, and peripheral white lesions (Figure 2).

**Figure 3:**
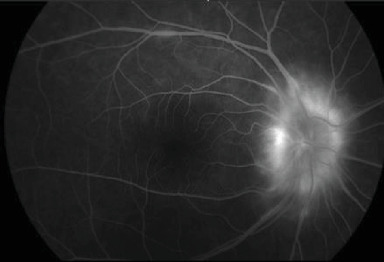
Initial FA in the RE showing optic disc and small vessel hyperfluorescence.

Initial posterior pole fluorescein angiography (FA) in the RE showed optic nerve and small vessel hyperfluorescence (Figure 3).

**Figure 4:**
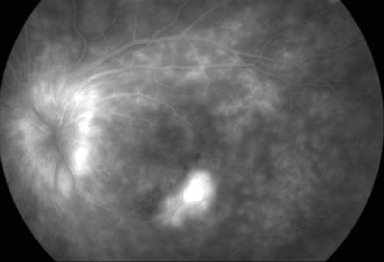
Initial FA in the LE showing optic disc and small vessel hyperfluorescence along with a CNVM.

Initial FA in the LE showed optic nerve and small vessel hyperfluorescence as well as a choroidal neovascular membrane (CNVM) (Figure 4). Indocyanine green (ICG) angiography was not available at the initial evaluation.

The patient received an initial diagnosis of bilateral ODE, intermediate uveitis RE, and posterior uveitis LE and was started on topical prednisolone acetate four times daily in both eyes (BE).

**Figure 5:**
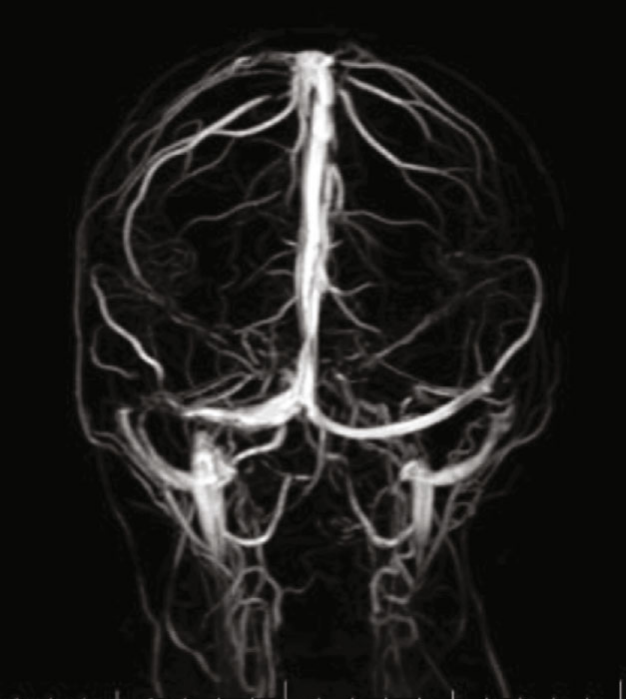
MRV showing narrowing of right and left transverse cerebral sinuses without thromboses.

Laboratory evaluation was initiated for infectious and inflammatory causes of uveitis and revealed the following normal results: Lyme IgM and IgG, Toxoplasma IgM and IgG, Quantiferon, RPR, TPA-ABS, ACE, ANCA, CBC, and ESR. Computed Tomography (CT) scan of the chest showed scattered calcified lymph nodes inconsistent with Sarcoidosis. Magnetic Resonance Imaging (MRI) of the brain and orbits was performed showing the bilateral ODE and revealed bilateral posterior globe flattening, a partially empty sella, amild distension of the perioptic nerve sheaths, but no optic nerve enhancement, white matter lesions, masses, or meningeal enhancement. Magnetic Resonance Venography (MRV) of the brain demonstrated narrowing of the right and left transverse sinuses without thromboses (Figure 5).

The initial lumbar puncture (LP) revealed elevated opening pressure of 35 cm H_2_0 and unremarkable CSF evaluation for protein, glucose, white and red blood cells, Zoster, Syphilis, and ACE. The patient was started on oral prednisone 60 mg daily for the uveitis, but treatment for the IICP was not initiated at this time for unclear reasons. The patient subsequently developed a thirty-pound weight gain and worsening headaches when attempting to quickly taper the oral prednisone, so this medication was gradually tapered over a few months. The vitreous inflammation did decrease in the LE, but visual acuity did not improve beyond 20/250 even after an intravitreal injection of bevacizumab for the CNVM.

Three months after initial presentation, while on prednisone 20 mg daily, the patient presented to the Emergency Department for worsening headaches. An LP at this time showed opening pressure of 45 cm H_2_0. Ocular ultrasonography was not available to confirm the diagnosis of papilledema at that visit. Neurology recommended initiating oral acetazolamide 1000 mg twice daily to treat the IICP as well as the headaches and tapered the prednisone over a few weeks.

**Figure 6:**
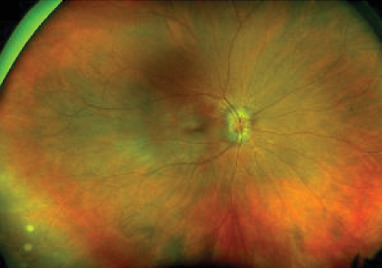
Widefield fundus photograph of the right eye showing optic disc edema and temporal atrophic spots.

**Figure 7:**
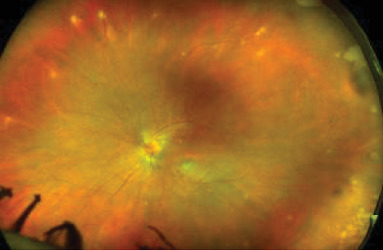
Widefield fundus photograph of the left eye showing optic disc edema, macular thickening with a gray macular lesion, and scattered atrophic peripheral spots.

Subsequent evaluation revealed best corrected visual acuity of 20/20 RE and 20/200 with pinhole LE. Intraocular pressure was normal in BE, and an afferent pupillary defect was identified in the LE. Slit lamp examination showed a trace anterior chamber cell in the RE and 2+ anterior chamber cell and flare with keratic precipitates in the LE. Dilated funduscopic exam showed a trace anterior vitreous cell, 360 degrees of ODE, and focal retinal atrophic spots in the RE (Figure 6) and 1+ anterior vitreous cell and haze, 360 degrees of ODE, cystoid macular edema (CME), a gray macular lesion, and scattered atrophic spots throughout the periphery of the LE (Figure 7).

**Figure 8:**
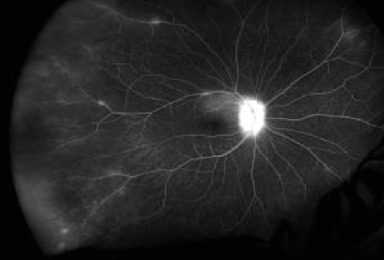
Widefield FA of the RE showing optic disc and segmental large vessel hyperfluorescence.

**Figure 9:**
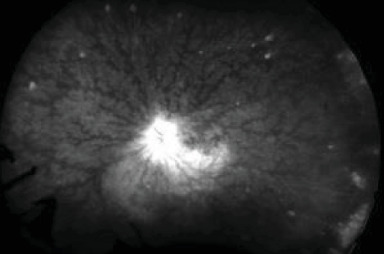
Widefield FA of the LE with disc, macular, and diffuse small vessel hyperfluorescence.

Widefield FA showed optic disc, small vessel, and segmental large vessel hyperfluorescence in the RE (Figure 8) and optic disc, macular, and diffuse small vessel hyperfluorescence LE (Figure 9).

**Figure 10:**
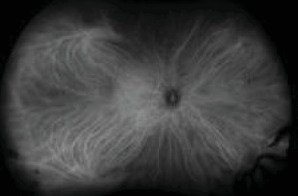
Widefield ICG showing peripheral hypocyanescent spots in the periphery of the RE.

**Figure 11:**
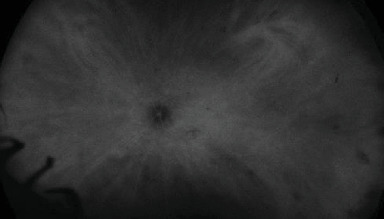
Widefield ICG showing a hypercyanescent spot in the macula and hypocyanescent spots in the periphery of the LE.

Widefield ICG angiography was also performed and revealed hypocyanescent spots in the periphery of the RE (Figure 10) and a hypercyanescent spot in the macula with peripheral hypocyanescent spots in the LE (Figure 11).

**Figure 12:**
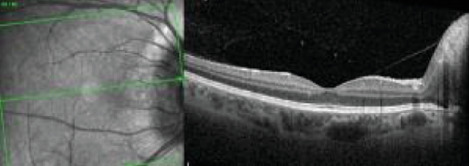
OCT of the macula showed peripapillary nerve fiber layer thickening with normal foveal contour in the RE.

**Figure 13:**
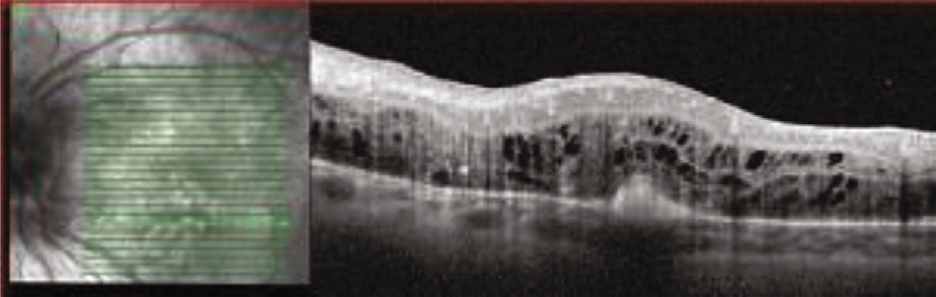
OCT of the LE with peripapillary nerve fiber layer thickening, cystoid macular edema with a focal pigment epithelial detachment.

Automated visual field testing showed an enlarged blind spot in the RE and a superior cecocentral defect in the LE. OCT of the macula showed peripapillary nerve fiber layer thickening with normal foveal contour in the RE (Figure 12) and peripapillary nerve fiber layer thickening, diffuse CME, and a pigment epithelial detachment in the LE (Figure 13). Retinal nerve fiber layer- (RNFL-) OCT was thick in both eyes.

**Figure 14:**
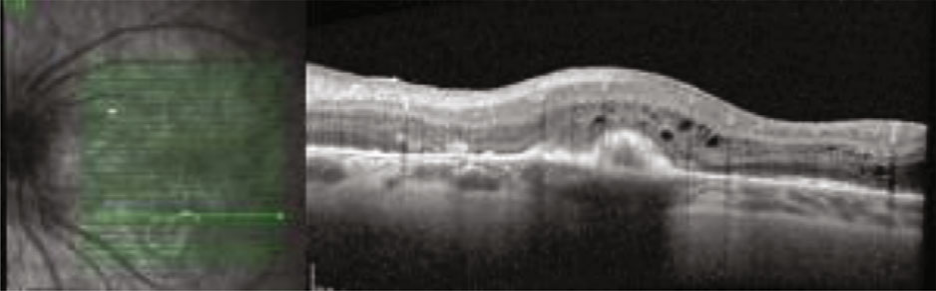
OCT of the LE three weeks after sub-Tenon's triamcinolone and intravitreal bevacizumab with improved macular edema.

The patient's diagnoses were refined to bilateral ODE, multifocal choroiditis, retinal vasculitis, and CME with a CNVM LE. As corticosteroids were relatively contraindicated given prior weight gain and IICP, the patient underwent additional safety lab testing for corticosteroid sparing immune suppression for the bilateral noninfectious uveitis. She received a periocular triamcinolone to the LE for the uveitis and CME and a second intravitreal bevacizumab injection in the LE for the inflammatory CNVM. There was improvement in acuity to 20/200 along with reduced macular edema (Figure 14).

Treatment for the IICP was continued with acetazolamide which was titrated up to 1250 mg twice daily with slow improvement in the bilateral ODE by RNFL. Oral corticosteroids were discouraged given the IICP. Steroid sparing immune suppressive therapy was strongly recommended given the bilateral uveitis, cystoid macular edema, and CNVM LE.

## 5. Discussion

Simultaneous posterior uveitis and papilledema have previously been reported by Margalit et al. who described a child with idiopathic intracranial hypertension and posterior uveitis [2] and Eken et al. who described a 16-year-old with bilateral posterior uveitis and papilledema from hydrocephalus secondary to a Chiari I malformation [3]. This is the known case of simultaneous posterior uveitis and IICP in an adult.

The evaluation of bilateral ODE, even in the presence of posterior uveitis, should include brain MRI-MRV and LP. When available, ocular ultrasonography can also be a useful noninvasive diagnostic tool for identifying optic nerve sheath distension as demonstrated by Vitiello et al. [1]. In this patient, ocular ultrasonography was not available to confirm the diagnosis of optic nerve sheath distension. Evaluation should also include visual field testing and RNFL for ODE. OCT as well as retinal fluorescein and choroidal indocyanine green angiography should be performed to assess uveitic disc edema associated with cystoid macular edema, retinal vascular inflammation, and multifocal choroiditis. Consultation with Rheumatology should be considered in such cases to evaluate for Behcet's disease, which can cause uveitic disc edema and papilledema from cerebral sinus thromboses [5]. As this patient did not have any oral or genital ulcers, she deferred the recommended Rheumatology consultation.

In this case, treatment of the uveitis with systemic corticosteroids partially improved the uveitis but caused weight gain and worsening headaches. This may have contributed to elevation of the intracranial pressure as demonstrated by higher opening pressure with the second LP. As treatment of uveitis with systemic corticosteroids has been shown to cause IICP in children [4], physicians must also be mindful of this association in adults. In this case, earlier initiation of oral acetazolamide should likely have commenced along with the systemic corticosteroids, although it is unclear if its delayed initiation will have a negative impact on the ultimate outcome. The periocular steroids for CME and intravitreal antivascular endothelial growth factor (VEGF) injections for the inflammatory CNVM were important for the acute sight threatening complications of uveitis, but the long-term focus will be on steroid sparing immune suppressive therapy in addition to therapy for the IICP.

It can be difficult to distinguish uveitic disc edema from papilledema. As such, monitoring uveitic disc edema and CME with angiography and OCT must continue, in addition to RNFL and visual field studies for papilledema as well as ocular ultrasonography if available. Treatment of uveitic disc edema should focus on reducing associated optic disc and retinal vascular leakage by angiography as well as reduction of CME and any CNVM with eye injections. Treatment of papilledema also needs to be optimized to preserve visual field and normalize retinal nerve fiber layer thickening. The ultimate goal of such multifaceted therapy is to prevent permanent vision loss as a result of optic disc and retinal atrophy. The prognosis in such cases of multifactorial optic nerve pathologies (uveitic disc edema and papilledema) as well as retinal vascular pathologies (diffuse small vessel leakage, CME, and CNVM) is unknown, but comprehensive monitoring is paramount to ensure optimal visual outcomes.

In summary, we present a case of simultaneous posterior bilateral uveitis and papilledema in an adult. Clinicians should consider neuroimaging and CSF analysis in such scenarios in addition to OCT and retinal angiography and ocular ultrasonography to monitor treatment of both uveitic disc edema and papilledema.

## Data Availability

The references and source image data used to support the findings of this study are available from the corresponding author upon request.
